# Undiagnosed Diabetes Mellitus and Its Predictors Among Socially Marginalized Menja Communities in Southwest Ethiopia

**DOI:** 10.3389/fpubh.2022.861627

**Published:** 2022-05-12

**Authors:** Ashenafi Assefa, Nigusie Shifera

**Affiliations:** ^1^Nursing Department, College of Medicine and Health Science, Mizan Tepi University, Mizan Teferi, Ethiopia; ^2^Department of Epidemiology and Biostatics, School of Public Health, Mizan Tepi University, Mizan Teferi, Ethiopia

**Keywords:** diabetes, DM, undiagnosed, Menja, marginalized

## Abstract

**Background:**

Diabetes mellitus (DM) is a metabolic disorder marked by a persistently high blood glucose level over a prolonged period of time linked to either defects in insulin secretion, insulin action, or both. It is responsible for 537 million adult cases and 6.7 million deaths in 2021. However, about half of the people with diabetes go undiagnosed. Low-income and socially disadvantaged communities are the most vulnerable to the disease. Despite this fact, nothing has been done among these communities, so this study aimed to assess the extent of undiagnosed diabetes and its predictors among the socially marginalized Menja communities of Southwest Ethiopia, 2021.

**Methods:**

A community-based cross-sectional study was done in the Menja communities from April 21/2021 to June 30/2021. The required sample size was calculated using the single population proportion formula and systematic sampling techniques were employed to select the households. Data were collected through face-to-face interviews utilizing an interviewer-administered questionnaire to collect socio-demographic and behavioral characteristics, and anthropometric measurements were taken from each participant. Diabetes was defined as participants who had an FBG ≥ 126 mg/dL or RBG > 200 mg/dL. The multivariate logistic regression model was used to identify the predictors of diabetes; adjusted OR with a 95% CI was computed to assess the strength of associations.

**Results:**

The prevalence of undiagnosed DM among the socially marginalized Menja communities was 14.7% [95% CI: (11.1–18.3)], and sex-specific prevalence was 16.8%, and 11.1% for men and women respectively. Factors like alcohol consumption (AOR = 3.0, 95% CI 1.49 to 6.05), family history of DM 4.4 (AOR = 4.37, 95% CI 2.04 to 9.35), lower vegetable consumption 3.5 (1.19–10.31) (AOR = 3.5, 95% CI 1.19 to 10.31), and less physical exercise 3.3 (AOR = 3.34, 95% CI 1.61 to 6.90) were the independent predictors that increase the risk of diabetes among Menja communities.

**Conclusion and Recommendations:**

Undiagnosed diabetes was high as compared to other settings. Alcohol use, family history of diabetes, vegetable consumption, and physical exercise were predictors of diabetes. Hence, the study suggests frequent screening and treatment for high-risk groups. Minimizing alcohol drinking, frequent vegetable consumption, and physical exercises were recommended measures for the prevention and control of DM among the population of Ethiopia.

## Introduction

Diabetes mellitus (DM) is a metabolic disorder marked by a persistently high blood glucose level over a prolonged period of time linked to either defects in insulin secretion, insulin action, or both (1, 2). Type 2 diabetes affects the majority of people, and nearly entirely occurs among adults (2). Around 537 million adults (20–79 years) worldwide have DM; 541 million have impaired glucose tolerance, putting them at high risk of developing the disease; this figure is expected to rise to 643 million by 2,030 and 784 million by 2,045, and more than four out of five (81%) adults live in low- and middle-income countries (3).

The disease prevalence has been rising more rapidly in low- and middle-income countries than in high-income countries, and the socially disadvantaged in any country are the most vulnerable to the disease (4, 5). In 2019, the International Diabetic Federation (IDF) Africa Region has approximately 19 million and 47 million persons (20–79) living with diabetes and impaired glucose tolerance, respectively. By 2,045, this number is expected to rise to 47 million people with diabetes and 110 million people with impaired glucose tolerance, putting them at a high risk of developing type 2 diabetes (6). According to the IDF, the prevalence of diabetes among Ethiopian adults is 3.2% (7).

Diabetes will be the seventh leading cause of death by 2,030, accounting for 6.7 million fatalities (1 every 5 s) (3, 8). It is expected to cost at least USD 966 billion in health expenditure in 2021, and this is a 316% increase over the last 15 years (3), In Africa about USD 9.5 billion was spent on healthcare for people with diabetes in 2019 (7). Furthermore, diabetes affected more than 21 million live births in the IDF Africa Region in 2013, accounting for 1 in every 9 live births (5, 7). Regular physical activity, a healthy body weight, a balanced diet, and avoiding tobacco use are among the most popular lifestyle changes that can help prevent this (8).

Poorly managed diabetes increases the risk of heart attacks and strokes by two to three times, as well as neuropathy (nerve damage), renal failure, foot ulcers, lower limb amputation, and 2.6 presence of global blindness in 2021 (9), Early identification and treatment are critical interventions for preventing diabetes complications and mortality, as well as for better treatment outcome (2), however, about half of diabetics go undiagnosed (3). Furthermore, consequences from undiagnosed diabetes have a significant impact on patients' quality of life, which may typically be avoided by early identifying risk factors (10).

According to the 2014 estimate, 179.2 million people worldwide have undiagnosed diabetes (2), the IDF Africa Region has the greatest rate of undiagnosed people of any of the IDF regions, with 60% of adults living with diabetes unaware of their condition (7). A systematic analysis from African countries revealed that the pooled prevalence of undiagnosed DM among adults was 3.85%, thus 4.43% in Eastern Africa; 4.72% in Western Africa; 4.27% in Northern Africa, and 1.46% in southern Africa respectively (11). In Ethiopia, various studies have revealed varying rates of undiagnosed diabetes. For example, research conducted in Bahr Dar, Dire Dawa, Gonder, and Dembia found that the prevalence of undiagnosed diabetes was 10.2, 6.2, 6.34, and 11.5% respectively (12–15).

Age, marital status, educational level, family history, overweight, lack of awareness of diabetes symptoms, alcohol consumption, waist circumference, history of hypertension, cigarette smoking, exercise, and low health-seeking behavior, such as lack of regular health check-ups, were all found to be independently associated with undiagnosed diabetes mellitus (12, 13, 15, 16). Even though these studies have been conducted in various parts of Ethiopia, nothing has been done among the socially marginalized communities. So, this study aimed to assess the prevalence of undiagnosed diabetes and its contributors among culturally marginalized communities of Menja in Southwest, Ethiopia, this helps to carry out evidence-based intervention for policymakers, governmental and non-governmental organizations.

## Methods and Materials

### Study Design, Setting, and Period

A community-based cross-sectional study was done in the Menja communities from April 21/2021 to June 30/2021. The Menja community is located 534 km from Addis Abeba, the capital of Ethiopia in the southwest direction. The Menja is a minority group living in pockets of the Kafa, and Bench Maji zone of the southwest region of Ethiopia. There is no census data on the Menja population because they live within the majority culture and are considered members of the majority ethnic group. Nevertheless, the Menja do have a separate identity. Menja community administration has 13 dispersed kebeles.

The Menja are hunters; most of their subsistence is derived from hunting wild animals such as colobus monkeys, porcupines, and wild boar, and from gathering and selling forest products such as firewood, charcoal, and honey. Farmers, who represent the majority of the society, consider themselves 'dirty' because of their different dietary habits, i.e., eating wild animals and those not ritually slaughtered. Indeed, the Menjo are disdained and discriminated against by the majority groups. The Menja population is physically different from farmers and other minorities as they are darker and shorter in stature and have curly hair, flat noses, and smaller foreheads. Until recently, the Menja population has not been the main focus of studies and listed substantive ethnographic data have been compiled about them.

### Population, Sample Size, and Sampling Method

The source population was all adults over the age of 18 who resided in the Menja community for more than 6 months, and the study population was adults over the age of 18 residing in eligible homes in randomly selected kebeles in the community. The single population proportion formula was used to compute the minimal sample size required for this research, with the following assumptions: Due use a lack of previous research in the study context, there was a 50% proportion of undiagnosed diabetes, a 95% confidence level, a 5% margin of error, and a 10% non-response rate, resulting in a total of 422 participants (17).

Based on the number of homes, the total sample size was proportionally allocated to each of the randomly selected kebeles. A systematic sampling approach was utilized to choose the households at every “K” interval (2,525/422 = 6) with a random starting point determined from inside the first 6 houses using the household record. For households with more than one eligible individual, the lottery technique was used to select only one person, but in the event of a household with no eligible individuals, the immediate next household (HH) was used.

### Data Collection Procedures and Measurement of Variables

After reviewing different kinds of literature, the English version data collection tool was prepared. The questionnaire was translated into the local language (Amharic and Benchgna) and back-translated into English to maintain consistency. The questionnaire was divided into four sections: socio-demographic, behavioral, or lifestyle, and clinical or anthropometric characteristics. The primary investigator recruited and trained four laboratory personnel (three data collectors and a supervisor) on the study's goal and how to collect blood samples. Face-to-face interviews utilizing an interviewer-administered questionnaire were used to collect socio-demographic and behavioral data.

#### Blood Pressure Measurement (BP)

It was measured on the right arm, the average was taken for two evaluations 5 min apart, and then Hypertension was defined as a systolic BP of ≥140 mmHg and diastolic BP of ≥ 90 mmHg (18).

#### Body Mass Index (BMI)

The formula for calculating body mass index (BMI) is weight in kilograms divided by height in meters squared. A BMI of <18.5 kg/m^2^, 18.5–24.9 kg/m^2^, >25–29.9 kg/m^2^, and ≥ 30 kg/m^2^ was recognized as underweight, normal, overweight, and obese respectively (19).

#### Current Substance Use

Using at least one of a specific substance (alcohol, khat, cigarette, and others) for non-medical purposes within the last 3 months according to the alcohol, smoking, and substance involvement screening tool (ASSIST) (20).

#### Ever Substance Use

Using at least one of any specific substances (alcohol, khat, cigarette, and others) for non-medical purposes at least once in a lifetime according to ASSIST (20).

#### Undiagnosed DM

Participants with undiagnosed diabetes (type 1 or type 2) with FBG ≥126 mg/dL or RBG ≥200 mg/dL were classified as those who had never had their blood sugar tested before and were not receiving DM medication at the time of the survey (21).

#### Physical Activity

The Global Physical Activity Questionnaire (GPAQ) was used to collect data, and activity levels were estimated using the cut-off points in the analytic guide (22).

#### Vegetable and Fruit Consumption

It was assessed using the WHO dietary assessment for vegetables and fruit, and then classified into two or fewer servings, one to three servings, and four to seven servings (23).

### Data Quality Control

To safeguard the quality of the data, a pre-test was done on 5% of the sample size at the neighboring Bachuma district having similar socio-cultural characteristics with study subjects, accordingly, the necessary modification was done. The questionnaire was initially prepared in English and then translated into a local language “Amharic and Benchigna”, and then back-translated into English to maintain its consistency. Moreover, the tool was assessed for its clearness, and the items that were problematic to answer were rephrased.

Four laboratory technicians received 4 days of training on questionnaire administration, physical measurement, and biomedical measurement techniques before the survey. The clinical data (family history of DM, hypertension, and ever checked glucose level) of the participants were also collected *via* interview. Laboratory personnel was organized on proper sample collection before beginning blood collection. Physical measurements were taken twice, and in some cases, three times, to reduce observer error. Data collectors were rotated to compare results in between the data collection. The glucometer apparatus and strips were verified for consistency in reference and test readings regularly.

The collected data completeness was checked manually before being entered into EpiData 3.1 and the necessary correction was made. The entire process of data collection was overseen by supervisors and the principal investigator. Supervisors reviewed the obtained data on a regular basis in the field. Data coding and cleaning were done by cross-checking to print out for possible errors. Missing values and outliers were checked through running descriptive analysis.

### Data Processing and Analysis

The data were cleaned, coded, and entered using Epi-Data version 3.1 and exported to SPSS version 24 for management and statically analysis. Descriptive statistics (means, medians, and percentages) were presented in texts, tables, and graphs. To identify candidate variables for the final model, bivariate logistic regression was used to assess the relationship of undiagnosed diabetes with each variable. Variables with value <0.25 were taken into multiple logistic regression analysis to identify independent predictors of DM.

The model was constructed using the backward likelihood ratio with 0.1 probability elimination. The resulting model's goodness of fit was assessed using the Hosmer-Lemeshow test of goodness of fit, with good fit defined as a value >0.05, and model classification accuracy subsequently assessed. Finally, independent predictors of DM were declared at a *p*-value < 0.05 cutoff point, and the strength of the association was assessed using AORs with their corresponding 95% confidence level.

## Results

### Socio-Demographic and Economic Characteristics

This survey had 415 people out of 422 who were chosen, with a response rate of 98.3%. The Mean age of the participants was 36.4 ± (8.7 SD) years. About 63.1% were men, 36.9% self-employed, and 42.7% of the participants had no formal education. More than half 53.5% were married and, a comparable proportion of 53.5% of the participants had an income < 2,500 Ethiopian birr (see Table 1).

**Table 1:** The socio-demographic and economic characteristics of the Menja community in Bench-Sheko zone Southwest Ethiopia, 2022, (*N* = 415).

**Variables**	**Category**	**Frequency (N)**	**Present (%)**
Sex	Male	262	63.1
	Female	153	36.9
Participants age	18–34	171	41.2
	35–54	160	38.6
	>55	84	20.2
Marital status	Single	130	31.3
	married	222	53.5
	divorced/separated	63	15.2
Participant religion	Protestant	124	29.9
	Orthodox	119	28.7
	Muslim	172	41.4
Educational level	Uneducated	177	42.7
	Primary & secondary	151	36.4
	Diploma and above	87	21.0
Occupation	unemployed	130	31.3
	self employed	153	36.9
	private employed	87	21.0
	government employed	45	10.8
Monthly Income	<2500 ETB	222	53.5
	≥ 2500 ETB	193	46.5

### Behavioral and Lifestyle Characteristics

Slightly < a quarter 21.7% of the participants were current smokers, with 9.2% of them smoking fewer than 5 numbers of sticks per day. About 40.7% had a history of alcohol drinking, and out of them, 19.5% had 1–3/days/month frequency intake. About 17.1% chewed khat, 41.2% had vegetable serving one to three times per week, and a similar proportion of 40.7% and 40.0% had a high (≥ 1,500 MET min) and medium (≥600 MET min) level of total physical activity, respectively (see Table 2).

**Table 2:** Behavioral and lifestyle characteristics of Menja community in South-west, Ethiopia, 2021 (*N* = 415).

		**Frequency**	**Present**
**Variables**	**Category**	**(*N*)**	**(%)**
Smoking	Yes	90	21.7
	No	325	78.3
Number of sticks	<5	38	9.2
per day	5–10	32	7.7
	>10	20	4.8
Alcohol drinking	Yes	169	40.7
	No	246	59.3
Frequency of	5–7 days/week	30	7.2
alcohol	1–4 days/week	26	6.3
drinking	1–3/days/month	81	19.5
	<1/month	32	7.7
Chat Chewing	Yes	71	17.1
	No	344	82.9
Total physical	High (≥ 1,500MET min)	169	40.7
activity (MET	Medium (≥600 MET minute)	141	40.0
minutes/week)	Low (<600 MET min)	105	25.3
Vegetable	Twice or fewer serving	148	35.7
consumption/	one to three serving	171	41.2
week	four to seven serving	96	23.1
Fruit	Twice or fewer serving	152	36.6
consumption/	one to three serving	224	54
week	four to seven serving	39	9.4

### Clinical and Anthropometric Measurements

One-quarter of the individuals (24.6%) had a BMI of 25 kg/m^2^ or higher, 17.1% had a family history of diabetes, and 11.6% had a previous history of hypertension diagnosis. About 14.9% of the participants had previously checked their blood glucose level, and slightly < a quarter (21.9% of the participants) were aware of diabetes symptoms, with polyphagia, polyuria, and polydipsia accounting for 35.2, 27.5, and 20.8% respectively (see Table 3).

**Table 3:** Clinical and anthropometric measurements of the participants, Menja community, South-west, Ethiopia, 2021 (*N* = 415).

		**Frequency**	**Present**
**Variables**	**Category**	**(N)**	**(%)**
Family history of	Yes	71	17.1
DM	No	344	82.9
Ever check blood	Yes	62	14.9
glucose level	No	353	85.1
Diagnosis of	Yes	48	11.6
hypertension	No	367	88.4
Know symptoms	Yes	91	21.9
DM	No	324	78.1
Body mass	under weight	67	16.1
index	normal	246	59.3
	over weight/obese	102	24.6
Abdominal obesity	Yes	85	20.5
	No	330	79.5

### Prevalence of Undiagnosed Diabetes

**Figure 1:**
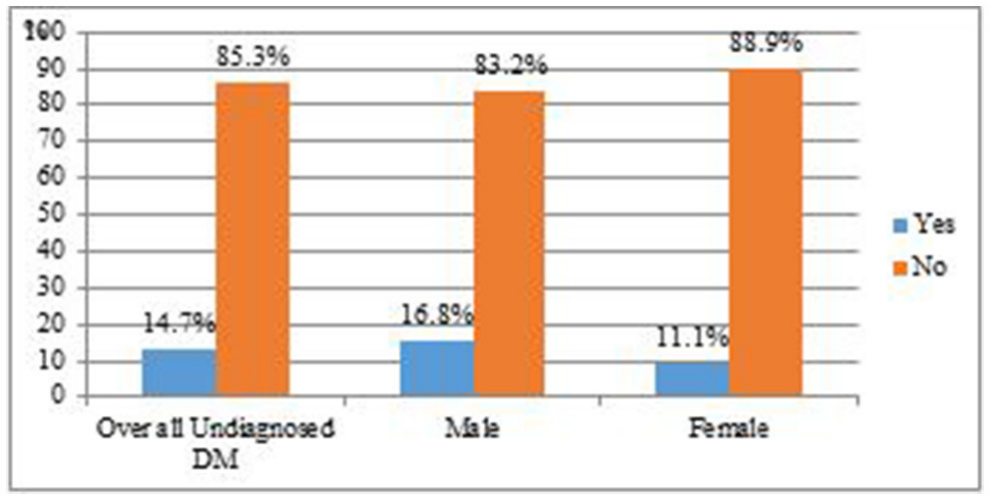
Prevalence of Diabetic Mellitus among Menja Community, South-west, Ethiopia, 2021 (*N* = 415).

The prevalence of undiagnosed DM was 14.7% [95% CI: (11.1–18.3)], with sex-specific prevalence being 16.8, and 11.1% for males and females, respectively (see Figure 1).

### Predictors of Undiagnosed Diabetes

During binary logistic regression, the candidate variables with a *p*-value < 0.25 were age, marital status, educational level, income, alcohol intake, chat use, physical activity, vegetable consumption, family history of diabetes mellitus, and body mass index. Alcohol use, vegetable consumption, and family history of diabetes were found to be independent predictors of undiagnosed DM after multivariate logistic regression with a *p-*value of < 0,005 cut-off thresholds.

The odds of having undiagnosed diabetes among individuals who drank alcohol were three times (AOR = 3.0, 95% CI 1.49 to 6.05) higher compared to those who did not drink. Similarly, those participants who had a family history of diabetes had 4.4 (AOR = 4.37, 95% CI 2.04 to 9.35) times higher odds of undiagnosed diabetes compared with those who had no such history. Moreover, individuals with two or fewer vegetable consumption/week were a 3.50 (1.19–10.31) (AOR = 3.5, 95% CI 1.19 to 10.31) higher risk of undiagnosed diabetes as compared to the higher frequency of vegetable consumption, and individuals with a low habit of physical exercise were 3.3 times (AOR=3.34, 95% CI 1.61 to 6.90) at higher risk of developing diabetes as compared to the counterparts (see Table 4).

**Table 4:** Multivariable analysis of different variables with undiagnosed DM in Menja community, Southwest Ethiopia, 2021 (*N* = 415).

**Variables**	**Undiagnosed DM**		**COR (95% CI)**	**AOR (95% CI)**	***P*** **-Value**
	**Yes (%)**	**No (%)**			
Age of the participant					
18–34	13 (21.3)	158 (44.6)	1	1	
35–54	27 (44.3)	133 (37.6)	2.46 (1.22–4.97)	2.33 (1.03–5.27)	0.052
>55	21 (34.4)	63 (17.8)	4.05 (1.91–8.58)	1.03 (0.36–2.94)	0.956
Marital Status					
Single	20 (32.8)	110 (31.1)	1	1	
Married	22 (36.1)	200 (56.5)	0.61 (0.32–1.16)	1.25 (0.35– 4.45)	0.726
Divorced/separated	19 (31.1)	44 (12.4)	2.34 (1.16–4.87)	2.86 (0.87–9.38)	0.083
Education level					
Uneducated	35 (57.4)	142 (40.1)	3.33 (1.34–8.25)	3.63 (0.89–14.75)	0.072
Primary & secondary	20 (32.8)	131 (37.0)	2.06 (0.79–5.35)	3.18 (0.77–13.07)	0.108
Diploma and above	6 (9.8)	81 (22.9)	1	1	
Monthly Income					
<2,500 ETB	40 (65.6)	182 (51.4)	1.80 (1.02–3.17)	2.18 (0.95–5.01)	0.067
≥ 2,500ETB	21 (34.4)	172 (48.6)	1	1	
Alcohol Drinking					
Yes	42 (68.9)	127 (35.9)	3.95 (2.20–7.08)	3.0 (1.49–6.05)	0.002^*^
No	19 (31.1)	227 (64.1)	1	1	
Khat use					
Yes	23 (37.7)	48 (13.6)	3.86 (2.12–7.03)	1.94 (.90–4.17)	0.12
No	38 (62.3)	306 (86.4)	1	1	
Total physical activity (MET minutes/week)					
High (≥ 1,500 MET minute)	12 (19.7)	157 (44.3)	1	1	
Medium (≥600 MET min)	25 (40.9)	116 (32.7)	2.8 (1.32–5.12)	1.62 (0.94–2.86)	0.062
Low (<600 MET min)	24 (39.4)	81 (22.8)	3.87 (2.19–7.21)	3.34 (1.61–6.90)	0.001^*^
Vegetable consumption/week					
Twice or fewer serving	35 (57.4)	113 (31.9)	3.94 (1.67–9.28)	3.50 (1.19–10.31)	0.022^*^
One to three serving	19 (31.1)	152 (42.9)	1.58 (0.64–3.93)	0.99 (0.334–2.96)	0.991
Four to seven serving	7 (11.5)	89 (25.1)	1	1	
Family History of DM					
Yes	27 (44.3)	44 (12.4)	5.59 (3.08–10.15)	4.37 (2.04–9.35)	<0.001^*^
No	34 (55.7)	310 (87.6)	1	1	
Body Mass Index					
<18kg/m^2^	6 (9.8)	61 (17.2)	1	1	
18–25 kg/m^2^	28 (45.9)	218 (61.6)	1.31 (0.52–3.29)	2.07 (0.55–7.76)	0.281
≥25 kg/m^2^	27 (44.3)	75 (21.2)	3.66 (1.42–9.43)	1.55 (0.35–6.84)	0.562

* *p value < 0.005, 1 = reference*.

## Discussions

The overall prevalence of undiagnosed diabetes was 14.7%, with sex-specific prevalence being 16.8, and 11.1% for men and women, respectively. Family history of DM, alcohol intake, vegetable consumption, and physical activity were all found to be independent predictors of undiagnosed diabetes. The prevalence stated above was consistent with studies done in Ethiopia; Addis Ababa (14.8%) (24), Sidama Zuria Woreda 12.4% (25), East Gojam (11.5%) (26), and Kenya 14% (27). However, our findings were higher than the studies in Canada 5.6% (28), Italy (10%) (29), Germany (8%) (30), Bahr Dar town (10.2%) (12), Bisheftu town (5%) (31), Dre Dawa (6.2%) (13), Gonder (6.34%) (14), Koladiba town north-west Ethiopia (2.3%) (15), Kuwait (6.9%) (32), and pooled estimate of the African population (5.37%) (33). This is the fact that the socially disadvantaged or marginalized communities in any country are the most vulnerable to the disease (2). However, the result was lower than that of an Indian study (15%) (34). Differences in socio-demographic characteristics, lifestyle or behavioral patterns, health-seeking behavior, and practice of routine screening for diabetes and other health conditions are expected to be the cause of the gap.

Participants who had a family history of diabetes had a 4.4 times higher risk of having undiagnosed diabetes as compared to those who had not. This statement is supported by a study done in Bahrdar town (12), East Gojam (26), and Koladiba town (15). This is a fact that can be attributed to the hereditary relationship between diabetes and obesity. Moreover, participants who had a history of alcohol drinking had three times higher odds of undiagnosed diabetes as compared to those who did not have such a history. This finding was supported by a study conducted in Ethiopia from Addis Abeba and Bisheftu study settings (24, 31). Alcohol may play a role in insufficient insulin release, diminished insulin binding, and suppression of intracellular signaling, all of which contribute to the development of insulin resistance (35).

This study also revealed that vegetable consumption is associated with undiagnosed diabetes. The odds of undiagnosed diabetes were 3.5 times higher among participants who had vegetables serving two or fewer/week compared to those who had vegetables serving four to seven/week. This report is supported by a study done on Gonder, this could be due to the fiber included in veggies, which helps to lower serum cholesterol while also reducing the release of sugar into the bloodstream, lowering fasting blood glucose levels (36). Moreover, individuals who had a low habit of physical exercise significantly increased the risks of diabetes. Individuals with a low habit of physical exercise were at 3.3 times higher risk of developing diabetes as compared to their counterparts, which is consistent with a study done in East Gojam, and the Southern region of Ethiopia, undiagnosed diabetes was higher risk among those participants with sedentary behavior (26, 37). Regular physical activity is beneficial not only in lowering insulin resistance and increasing insulin production but also in lowering the risk of cardiovascular disease and obesity in diabetic individuals (38).

## Conclusion and Recommendations

The prevalence of undiagnosed diabetes was high in the study setting. Alcohol use, family history, vegetable consumption, and physical exercise were the independent predictors of undiagnosed diabetes. As a result, the study recommends frequent screening and treatment for those with a family history of the disease, and raising awareness about the disease is recommended to aid in early detection and treatment. Minimizing alcohol drinking, frequent vegetable consumption, and physical exercise were recommended measures for the prevention and control of DM. Moreover, governmental and non-governmental organizations that are working in the area should give attention to these communities while providing diabetes-related services.

### Strengths and Limitations of the Study

The strengths of this study were that it was community-based and included sufficient samples. The finding could be useful as a starting point for community screening programs for high-risk groups. However, the study has limitations in that it did not show the actual overall magnitude of diabetes, as known patients with diabetes were omitted. Furthermore, the study's cross-sectional design makes it difficult to demonstrate cause-and-effect linkages between the dependent and independent variables.

## Data Availability Statement

The raw data supporting the conclusions of this article will be made available by the authors, without undue reservation.

## Ethics Statement

The studies involving human participants were reviewed and approved by Mizan Tepi University. The patients/participants provided their written informed consent to participate in this study.

## Author Contributions

AA and NS had a significant contribution to this research in the conception and design of the study, data acquisition, data analysis, and interpretation, drafting and revising of the article, agreed to submit to the current journal, gave final approval of the version to be published, and agree to be accountable for all aspects of the work. Both authors contributed to the article and approved the submitted version.

## Conflict of Interest

The authors declare that the research was conducted in the absence of any commercial or financial relationships that could be construed as a potential conflict of interest.

## Publisher's Note

All claims expressed in this article are solely those of the authors and do not necessarily represent those of their affiliated organizations, or those of the publisher, the editors and the reviewers. Any product that may be evaluated in this article, or claim that may be made by its manufacturer, is not guaranteed or endorsed by the publisher.

## Abbreviations

AOR: adjusted odds ratio
BMI: body mass index
BP: blood pressure
CI: confidence interval
COR: crude odd ratio
DM: diabetes mellitus
FBS: fasting blood sugar
GDM: gestational diabetes mellitus
HH: household
IDF: International Diabetes Federation
SD: standard deviation
WHO: World Health Organization.
